# Ribotype 027 *Clostridium difficile* infections with measurable stool toxin have increased lactoferrin and are associated with a higher mortality

**DOI:** 10.1007/s10096-013-2043-1

**Published:** 2014-01-22

**Authors:** J. H. Boone, L. R. Archbald-Pannone, K. N. Wickham, R. J. Carman, R. L. Guerrant, C. T. Franck, D. M. Lyerly

**Affiliations:** 1 Research and Development, TechLab, Inc., 2001 Kraft Drive, Blacksburg, VA 24060 USA; 2 Department of Internal Medicine, University of Virginia, Charlottesville, VA 22908 USA; 3 Department of Statistics, Virginia Tech, Blacksburg, VA 24061 USA

## Abstract

We evaluated clinical and diagnostic indicators of severe *C. difficile* infection (CDI) and their association with poor clinical outcome. A total of 210 patients positive according to PCR (toxin B: *tcd*B) were included, with patients having a median age of 62 years and a Charlson co-morbidity index (CI) score of 5. Ninety-one percent (*n* = 191) were positive by toxigenic culture and 61 % (*n* = 129) had stool toxin. Toxin-positive patients had significantly higher fecal lactoferrin (mean 316 μg/g versus 106 μg/g stool; *p* < 0.0001). Forty percent of patients (*n* = 85) were infected with ribotype 027 and significantly more of these patients had measurable stool toxin (79 % vs. 50 %; *p* < 0.0001). The mean fecal lactoferrin was significantly higher for toxin-positive 027 CDI compared with the 027 toxin-negative group (317 vs 60 μg/g; *p* = 0.0014). Ribotype 027 CDI with stool toxin showed a higher all-cause, 100-day mortality compared with non-027 with stool toxin (36 % vs 18 %; *p* = 0.017). Logistic regression univariate analysis for odds ratio (OR) and *p* values revealed that age (OR = 1.1), intensive care unit treatment (OR = 2.7), CI (OR = 1.2), 027 CDI (OR = 2.1), white blood cell count (OR = 1.0), albumin level (OR = 0.1), and stool toxin-positive 027 CDI (OR = 2.5) were significantly associated with 100-day mortality (*p* < 0.05). In conclusion, CDI PCR-positive patients with 027 infection and stool toxin have increased lactoferrin and are at an increased risk of death.

## Introduction

*C. difficile* infection (CDI) is a common complication for hospitalized patients. Identifying and appropriately treating patients who are more likely to suffer poor outcomes from this infection would improve clinical outcome and decrease healthcare costs. In the United States, the mortality rate from CDI has increased from 5.7 deaths per million people in 1999 to 23.7 deaths per million in 2004 [1]. The emergence of ribotype 027 BI/NAP1 in the early 2000s has led to outbreaks of CDI with more severe disease and an increased death rate in hospitalized patients [2–9]. The increased severity and transmission seen with the ribotype 027 has correlated with a combination of elevated early in vitro toxin production, increased sporulation, efficient germination, and resistance to fluoroquinolones [10–15]. Even though this strain has continued to increase in prevalence, the association with more severe disease is not consistent among studies, raising questions as to what cofactors contribute to severe CDI and worse outcomes [16–20]. Further studies are needed to target more accurately the combination of variables that contribute to severe infections and poor outcomes following CDI.

Laboratory diagnosis of CDI has changed over recent years resulting in molecular testing for the toxin genes (*tcdA* and *tcdB*) becoming more common in the United States, while the detection of toxin continues to be the test of choice in Europe [21, 22]. Both methods offer advantages. Molecular assays are highly sensitive showing between a true-positive rate of 75 and 85 % for toxigenic culture in many comparative studies [21]. GDH detection and toxin enzyme-linked immunoassay (EIA) tests, on the other hand, indicate the presence of the actively growing vegetative toxigenic *C. difficile* and correlate with worse outcome [23, 24]. These different tests have generated an increasing need for defined clinical criteria and host biomarkers that accurately identify patients who require aggressive treatment.

Various clinical criteria and blood biomarkers have been associated with increased CDI severity and poor clinical outcome. These factors include age, medical co-morbidities, clinical symptoms of fever and abdominal pain, increased peripheral white blood cell count (WBC), elevated serum creatinine, and low serum albumin [25– 32]. *C. difficile* toxins act locally to create intense intestinal inflammation that can be seen by colonoscopy or radiographic imaging, or measured in stool by fecal leucocytes or elevated fecal lactoferrin [19, 33–36]. More recently, fecal lactoferrin and fecal IL-8, both biomarkers of inflammation, have been shown to identify more severe cases of CDI [37–40]. In our study, we evaluated a novel combination of patient characteristics, clinical laboratory results, and fecal parameters with the infecting ribotype to evaluate their relationship with poor clinical outcome, as measured by 100-day, all-cause mortality for hospitalized patients positive for *C. difficile* according to PCR.

## Materials and methods

### Study subjects

Sequential in-patients from May 2010 to August 2011 with a clinical diagnosis of CDI and positive for *tcdB* according to PCR (BD GeneOhm C diff Assay, Becton Dickinson, Franklin Lakes, NJ, USA, or Xpert *C. difficile*, Cepheid, Sunnyvale, CA, USA) were identified according to the protocol (HSR-IRB #13630, NIH/NIAID 5K23A1074681). Patients were deemed eligible if hospitalized at the time of CDI diagnosis, ≥ 18 years old, no recent history of chronic diarrhea (≥ 4 weeks), and adequate volume of stool for subsequent testing. Enrolled subjects were followed for clinical outcome by review of medical records and telephone contact following diagnosis.

### Chart reviews

Reviews of medical records were carried out by participating clinical research team members to record patient demographics, medical co-morbidities, and clinically obtained blood and serum laboratory results (peripheral white blood cell count, serum albumin, and serum creatinine) at the time of CDI diagnosis. During hospitalization, all-cause treatment in the intensive care unit (ICU) and 100-day, all-cause mortality were recorded. An age-unadjusted Charlson co-morbidity Index score for assessing co-morbid disease was calculated for each patient, as described previously [41, 42].

### Toxin detection

*C. difficile* testing was done at the hospital clinical microbiology laboratory using *tcdB* PCR (BD GeneOhm C diff Assay, Becton Dickinson, or Xpert *C. difficile*, Cepheid) according to the manufacturer’s instructions. A fecal specimen from eligible *tcdB*-positive subjects on the day of CDI diagnosis was frozen at −80 °C and shipped to TechLab, Blacksburg, VA, USA, for biomarker testing and toxigenic culture, as well as PCR ribotyping. Stool toxin B was detected by tissue culture using the C. DIFFICILE TOX-B Test (TechLab^®^ Inc., Blacksburg, VA) including a neutral goat serum control as described by Boone et al. [37]

### Fecal lactoferrin analysis

The amount of fecal lactoferrin (μg/g stool) was quantitatively determined using the IBD-SCAN® test, according to the package insert (TechLab®).

### Bacterial culture and ribotyping

Toxigenic culture was obtained using the ethanol shock method previously described by Boone et al. [43] and *C. difficile* isolates were evaluated for PCR ribotype using the procedure developed by Stubbs et al. [44].

### Statistical analysis

A summary was tabulated to describe patient demographic and diagnostic characteristics. In cases where a skew was above 1.5 in magnitude (i.e. greater than 1.5 or less than -1.5), medians and ranges were reported. Otherwise, means and standard deviations (sd) were provided. Significance testing for percentage differences was based on Fisher’s Exact Test and Wilcoxon rank sum test for continuous variables. Logistic regression was used to assess the bivariate relationships between 100-day all-cause mortality and the available clinical and lab results. A predictive model was formed in the logistic regression framework using the Bayesian Information Criteria (BIC) to determine a parsimonious subset of variables which were highly predictive of 100-day all-cause mortality. Predictive accuracy was assessed using the concordance index, or c-statistic, which measures the tradeoff between true positive and false positive rates across the range of model predictions. The c-statistic is defined between zero and one. A c-statistic of 0.5 corresponds to a diagnostic rule as effective as a coin flip, and higher c-statistics are better. A significant difference was defined as having a *p*-value of ≤ 0.05.

## Results

A total of 210 adult hospitalized patients who tested positive for *C. difficile* according to PCR (for *tcdB*) were enrolled. The cohort was 50 % (*n* = 105) female, mean age was 60.4 (sd = 16.8) years and 44 % (*n* = 93) were ≥ 65 years old. The mean Charlson co-morbidity index score was 5 (sd = 3) points and the most frequently reported co-morbid conditions included 46 % (*n* = 96) having moderate to severe renal failure and 34 % (*n* = 72) suffering with dementia. A 63 % majority of subjects (*n* = 132) had a score of > 3 points using the Charlson co-morbidity index. Ninety-five subjects (45 %) were treated in the intensive care unit (ICU) during this hospitalization and 50 subjects (24 %) died within 100 days of CDI diagnosis, with a mean of 36 (sd = 29) days until death. The recorded cause of death for the 50 subjects included 15 (30 %) unknown, 14 (28 %) sepsis with 2 documented as CDI sepsis, 6 (12 %) malignancy, 5 (10 %) respiratory failure, 4 (8 %) liver disease, 4 (8 %) congestive heart failure, and 2 (4 %) infection. The patient demographics and clinical characteristics are listed in Table 1.

**Table 1:** Patient characteristics. Mean and standard deviation are reported for continuous variables. The skew for all continuous variables was below 1.5 in magnitude. Sample sizes ranged from 206 to 210 for these measures

Patient characteristics	Frequency	Percent
Age in years, mean = 60.4, sd = 16.8		
<65	117	55.7
≥65	93	44.3
Male gender	105	50
Charlson index, mean = 4.9, sd = 2.9		
0	7	3.3
1–3	71	33.8
4–6	79	37.6
>6	53	25.2
Comorbidities		
Myocardial infarction	54	25.7
Congestive heart failure	51	24.3
Peripheral vascular disease	24	11.4
Cerebrovascular disease	31	14.8
Dementia/delirium	72	34.3
Connective tissue disorder	16	7.6
Chronic pulmonary dysfunction	49	23.3
Gastric ulcer disease	32	15.2
Mild liver disease	17	8.1
Diabetes (no end organ damage)	37	17.6
Hemiplegia	16	7.7
Moderate-severe renal disease	96	46.2
Diabetes with end organ damage	28	13.4
Any tumor	60	28.6
Leukemia	5	2.4
Lymphoma	7	3.4
Moderate to severe liver disease	28	13.5
Metastatic solid tumor	20	9.7
AIDS	3	1.4
Intensive care unit	95	45.2
100-day mortality	50	23.8

Based on stool studies for CDI, 91 % of subjects (*n* = 191) were positive by toxigenic culture, and stool toxin, as determined by tissue culture, was detected in 61 % (*n* = 129). The top 5 most prevalent toxigenic ribotypes in this patient cohort were ribotype 027 at 40 % (*n* = 85), ribotype 014 at 10 % (*n* = 20), ribotype 106 at 6 % (*n* = 12), ribotype 056 at 6 % (*n* = 12), and ribotype 053 at 5 % (*n* = 9).

Systemic and intestinal inflammatory biomarkers and indicators of *C. difficile* disease were measured in blood and fecal specimens to assess disease severity and the overall medical condition of the patient (Table 2). Thirty-two percent of subjects (*n* = 67) had WBC > 15 × 10^9^/L. Serum albumin was measured in 77 % of enrolled subjects (*n* = 162) and 20 % of these subjects (*n* = 33) had low serum albumin levels (≤ 2.5 g/dL). Creatinine levels were ≥ 1.5 mg/dL for 28 % (*n* = 59) of subjects. A majority of the cohort (77 %; *n* = 161) had elevated lactoferrin (≥7.25 μg/g stool) and nearly half (37 %; *n* = 78) had lactoferrin levels over 100 μg/g stool. The frequency of each diagnostic parameter for the study cohort is listed in Table 2 and the reported means or medians depending on the skew are listed in Table 3.

**Table 2:** Diagnostic parameters. Frequencies and percentages are reported for categorical variables. Sample sizes range between 207 and 210 except for albumin, which has a sample size of *n* = 162

Diagnostic parameters	Frequency (%)
PCR +	210 (100)
Tissue culture +	129 (61.4)
Toxigenic culture +	191 (91.0)
Ribotype 027 CDI	85 (40.5)
White blood cell count	
<10 × 10^9^/L	89 (42.6)
10,000–15,000 × 10^9^/L	52 (24.9)
>15 × 10^9^/L	68 (32.5)
Lactoferrin	
<7.25, μg/g	49 (23.3)
7.25–100 μg/g	83 (39.5)
>100 μg/g	78 (37.1)
Albumin level	
<2.5 g/dL	33 (20.4)
≥2.5 g/dL	129 (79.6)
Creatinine	
<1.5 mg/dL	148 (71.5)
≥1.5 mg/dL	59 (28.5)

**Table 3:** Diagnostic parameters. Skew, median, minimum, and maximum are reported for continuous variables with skew above a magnitude of 1.5. Mean and standard deviation are reported for continuous variables with skew less than or equal to 1.5 in magnitude

Diagnostic parameters	Summary statistics
White blood cell count, skew, median (minimum, maximum)	1.7, 11.4 (0.2, 53.9)
Lactoferrin, skew, median (minimum, maximum)	3.3, 48.5 (0, 2,823)
Albumin level, mean (sd)	3.0 (0.65)
Creatinine, skew, median (minimum, maximum)	3.0, 0.9 (0.4, 9.9)

**Fig. 1:**
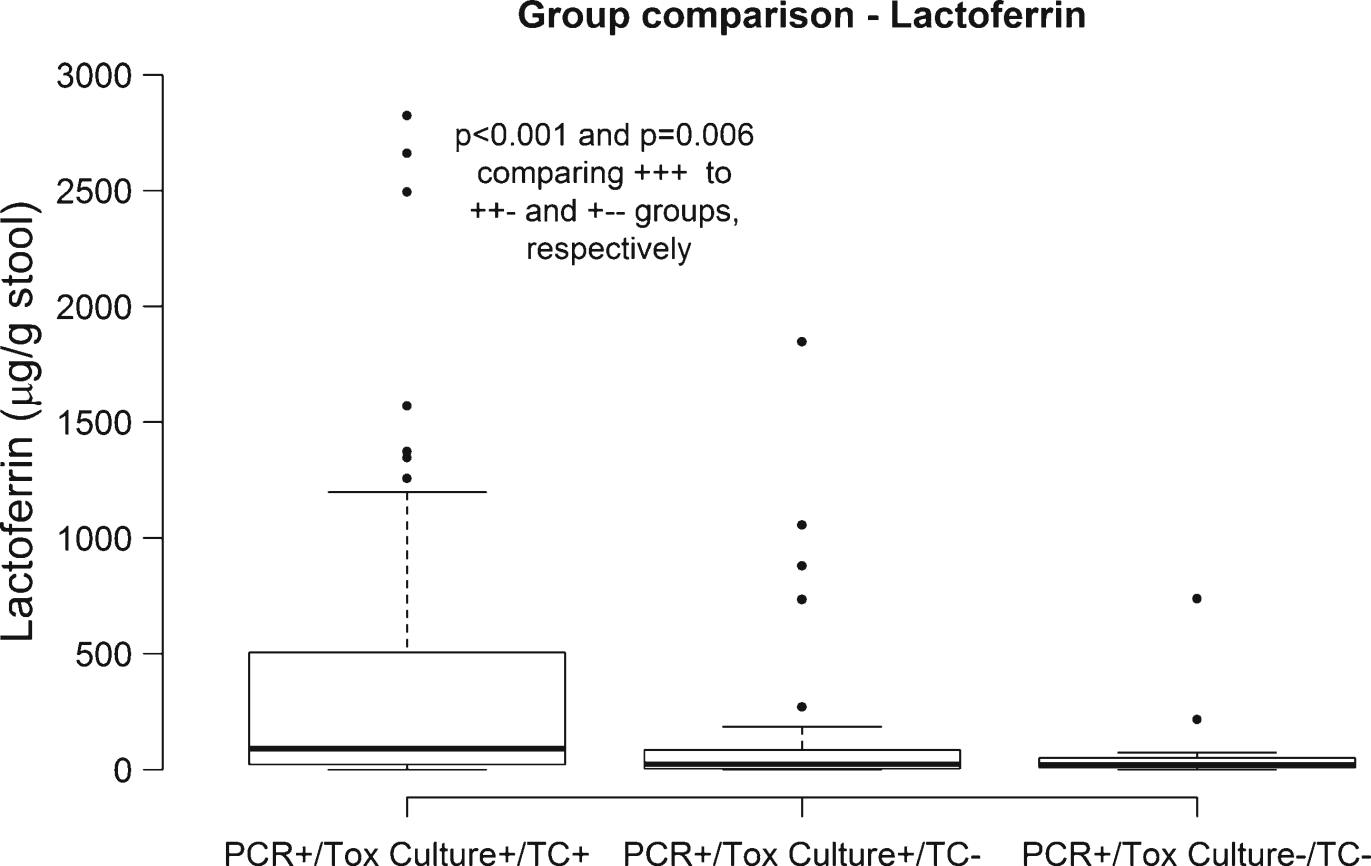
Comparison of lactoferrin median levels between diagnostic groups. Medians are noted with a *bold line* and *bars* and *dots* show lower and upper quartiles. Units for lactoferrin are shown in parenthesis. *p* values not included in the plot are above 0.05

**Fig. 2:**
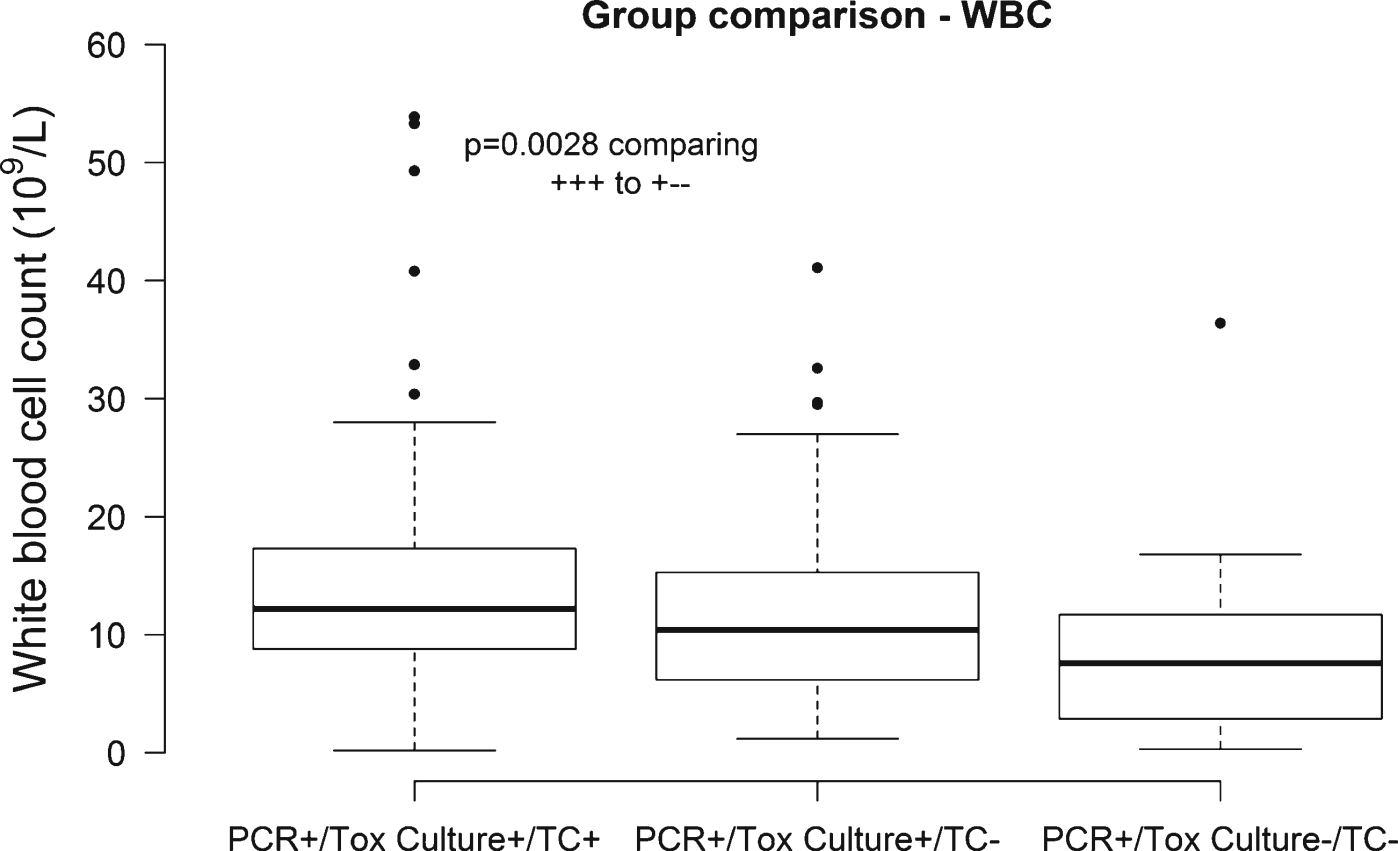
Comparison of white blood cell (WBC) median counts in the diagnostic groups. Medians are noted with a *bold line* and, *bars* and *dots* show lower and upper quartiles. Units for WBC count are shown in parenthesis *p* values not included in the plot are above 0.05

Each subject, all of whom were PCR + for *tcdB*, was placed in a group based on positive (+) or negative (−) toxigenic culture (tox culture) and stool toxin (tissue culture; TC). The diagnostic categories were: group 1 tox culture  (+)  tissue culture  (+) (61 %; *n* = 129), group 2 tox culture  (+ ) tissue culture (−) (30 %; *n* = 62), and group 3 tox culture (−) tissue culture (−) (9 %; *n* = 19). Using this grouping, median fecal lactoferrin was significantly higher for group 1 subjects (90 μg/g stool) compared with groups 2 (24 μg/g stool) and 3 (20 μg/g stool; Wilcoxon rank sum *p* < 0.0001 and *p* = 0.006 respectively; Fig. 1). WBC median counts differed significantly between group 1 (12.2 × 10^9^/L) and 3 (7.6 × 10^9^/L; *p* = 0.003; Fig. 2). There were no differences observed among the three groups for serum albumin and serum creatinine.

A series of univariate logistic regression analyses were performed to assess the association with all-cause mortality within 100 days of CDI diagnosis. Table 4 shows odds ratios, confidence intervals, and *p* values for the association between predictive variables and 100-day mortality. Age (*p* < 0.0001), Charlson co-morbidity index (*p* = 0.0002), ICU treatment (*p* = 0.0027), 027 infection (*p* = 0.0271), WBC count (*p* = 0.0283), albumin (*p* = 0.0199) ,and the combination of 027 infection with stool toxin (*p* = 0.0059) showed significant associations with increased mortality. The variables in Table 4 were then used as candidate predictors of 100-day, all-cause mortality in a logistic regression model excluding albumin levels, which has substantially fewer observations than the other variables. A model selection was performed by enumerating all possible models formed from the ten candidate predictors, and the model with the lowest value of Bayesian Information Criteria (BIC) was chosen. This model selection revealed that age (0 < 0.001), ICU treatment (*p* = 0.003), Charlson co-morbidity index (*p* < 0.001) formed a model with the best trade-off between overall predictive ability of death and complexity as measured by BIC. The true-positive trade-off of this model was good, as measured by a C-statistic =0.774, and supports these clinical characteristics as being significant in a high-risk patient population. When considering biomarkers for CDI, among the subjects who were stool toxin-positive, the subjects with 027 CDI had a significantly higher mortality rate (36 %; *n* = 24) than that (18 %; *n* = 11) for subjects with non027 infections (*p* = 0.017). The remaining groups, 027- and non-027-infected subjects with no measurable stool toxin had mortality rates of 17 % (*n* = 18) vs 19 % (*n* = 62) respectively.

**Table 4:** Univariate analysis for a combination of patient characteristics and fecal and blood biomarkers for an association with increased mortality. *p* values ≥ 0.05 are considered significant

Effect	Odds ratio est	Lower CL	Upper CL	Prob Chi Sq	*n*
Age	1.051	1.025	1.077	<0.0001	210
Sex	0.729	0.385	1.381	0.3319	210
Charlson index	1.235	1.105	1.38	0.0002	210
ICU	2.737	1.416	5.29	0.0027	210
027 CDI	2.064	1.086	3.926	0.0271	210
WBC	1.038	1.004	1.073	0.0283	209
Lactoferrin	1.000	1.000	1.001	0.1407	210
Albumin	0.503	0.282	0.897	0.0199	162
Creatinine	1.019	0.828	1.254	0.8577	207
027 and stool toxin +	2.512	1.304	4.839	0.0059	210
All stool toxin +	1.638	0.828	3.239	0.1561	210

## Discussion

In our study cohort, hospitalized patients with PCR-positive *C. difficile* infection having measurable stool toxin had significantly higher fecal lactoferrin and peripheral WBC, as indicators of more inflammation and severe CDI. Univariate analysis showed that age, Charlson co-morbidity index, ICU treatment, increased peripheral WBC, elevated lactoferrin, decreased albumin, and elevated creatinine were significantly associated with death within 100 days of CDI diagnosis. Multiple studies have shown the link between stool toxin/ inflammation and severe CDI/mortality [23, 24, 37–39, 40, 45, 46]. In a study by Baker et al. [46], patients with CDI who were positive for stool toxin according to EIA had a mortality rate of 38 % compared with 11 % for patients who were PCR-positive/EIA-negative. They also showed that patients with stool toxin were also more likely to suffer with prolonged diarrhea (2; *p* < 0.001). In another study by Bloomfield et al. [26], blood parameters including WBC, serum albumin, creatinine, and C-reactive protein were assessed as risk factors for 30-day all-cause mortality in patients who were positive for stool toxin [26]. Based on a multivariate model, WBC >15 × 10^9^/L and serum albumin levels <2.5 g/dL were significantly associated with mortality. The patient cohort included in the study by Bloomfield et al. [26] was similar to our cohort and included hospitalized patients with a median age of 74 years, with 78 % having a Charlson co-morbidity index score >3. Patients in our study were also older, with 44 % of patients being over 65 years and 71 % having a Charlson co-morbidity index score of >3. Our results support the clinical association of having stool toxin with risk of death in older CDI patients and further show that these patients suffer more intestinal inflammation, as indicated by higher lactoferrin levels. In addition, we determined that patients infected with ribotype 027 in combination with stool toxin and higher lactoferrin had a significantly higher mortality rate than toxin-negative 027-infected patients and all non-027 groups. Our logistic regression model using age, ICU treatment, and Charlson co-morbidity index was also successful at predicting death for this high-risk cohort (C-statistic = 0.774). These findings contribute to the growing evidence that supports the clinical utility for both PCR and stool toxin detection in identifying patients with *C. difficile* disease for appropriate treatment. We also show results that confirm the significance for identifying the infecting ribotype in a high-risk population and raise an important question on whether this information would modify treatment and improve patient outcome.

Further studies are needed to determine the effects of toxin levels produced by ribotypes like ribotype 027 and the potential link between circulating toxin and mortality as described recently in an animal model study by Kuehne et al. [47]. In this recent study, stain R20291, which produces toxins A and B along with binary toxin (CDT), was mutated to have combinations of toxins A and B, and CDT expression to assess the role of CDT in causing disease. Interestingly, the mutant strain only expressing CDT killed 3 out of 9 infected hamsters. Disease presentation in these animals was atypical and only included wet tail and evidence of small bowel inflammation. The authors went on to conclude that these results suggest the role of increased virulence for *C. difficile* strains like ribotype 027 with binary toxin [47]. In another study by Koon et al. [48], human colonic biopsies and isolated human monocytes were used to show induced expression of the inflammatory cytokines TNFα and IL-β by toxins A and B. In addition, infusions with monoclonal antibodies against toxin A and B were used to reduce the cytokine levels and protect epithelial cells from damage in human colonic tissue [48]. Our findings, along with these recent studies, further confirm the role of *C. difficile* toxin in causing intestinal inflammation and tissue damage, leading to a higher risk of death. In our patient cohort, 027-infected patients may have higher amounts of stool toxin, possibly causing systemic disease and death. Further studies are needed to determine the effects of toxin levels produced by ribotypes like ribotype 027 and the potential link between circulating toxin and mortality.

Our study confirms the clinical relevance of detecting stool toxin in patients who are PCR-positive for *C. difficile*. Patients with stool toxin, elevated lactoferrin, and infected with ribotype 027 are at an increased risk of death. A combination of clinical criteria, blood and fecal biomarkers for disease severity, and identifying the infecting ribotype should be considered in the evaluation of older, sicker patients with suspected CDI.
